# Body Composition Changes after a Weight Loss Intervention: A 3-Year Follow-Up Study

**DOI:** 10.3390/nu13010164

**Published:** 2021-01-07

**Authors:** Miguel A. Rojo-Tirado, Pedro J. Benito, Jonatan R. Ruiz, Francisco B. Ortega, Blanca Romero-Moraleda, Javier Butragueño, Laura M. Bermejo, Eliane A. Castro, Carmen Gómez-Candela

**Affiliations:** 1 LFE Research Group, Department of Health and Human Performance, Facultad de Ciencias de la Actividad Física y del Deporte, Universidad Politécnica de Madrid, 28040 Madrid, Spain; ma.rojo@upm.es (M.A.R.-T.); javier.butragueno@upm.es (J.B.); 2 Department of Physical Education and Sport, School of Sport Sciences, University of Granada, 52005 Granada, Spain; ruizj@ugr.es (J.R.R.); ortegaf@ugr.es (F.B.O.); 3 Applied Biomechanics and Sports Technology Research Group, Departamento de Educación Física, Deporte y Motricidad Human, Autonomous University of Madrid, 28049 Madrid, Spain; blanca.romero@uam.es; 4 Real Federación Española de Fútbol, Calle Ramón y Cajal s/n, 28232 Las Rozas de Madrid, Madrid, Spain; 5 Nutrition Department, Hospital La Paz Health Research Institute (IdiPAZ), La Paz University Hospital, 28046 Madrid, Spain; mlbermej@ucm.es (L.M.B.); carmengomezcandela@telefonica.net (C.G.-C.); 6 Department of Sports Sciences and Physical Conditioning, Faculty of Education, Universidad Católica de la Santísima Concepción, 4090541 Concepción, Chile; elianeaparecidacastro@gmail.com

**Keywords:** body weight, exercise, intervention study, nutrition, obesity

## Abstract

Studies comparing different types of exercise-based interventions have not shown a consistent effect of training on long-term weight maintenance. The aim of this study was to compare the effects of exercise modalities combined with diet intervention on body composition immediately after intervention and at 3 years’ follow-up in overweight and obese adults. Two-hundred thirty-nine people (107 men) participated in a 6-month diet and exercise-based intervention, split into four randomly assigned groups: strength group (S), endurance group (E), combined strength and endurance group (SE), and control group (C). The body composition measurements took place on the first week before the start of training and after 22 weeks of training. In addition, a third measurement took place 3 years after the intervention period. A significant interaction effect (group × time) (*p* = 0.017) was observed for the fat mass percentage. It significantly decreased by 5.48 ± 0.65%, 5.30 ± 0.65%, 7.04 ± 0.72%, and 4.86 ± 0.65% at post-intervention for S, E, SE, and C, respectively. Three years after the intervention, the fat mass percentage returned to values similar to the baseline, except for the combined strength and endurance group, where it remained lower than the value at pre-intervention (*p* < 0.05). However, no significant interaction was discovered for the rest of the studied outcomes, neither at post-intervention nor 3 years later. The combined strength and endurance group was the only group that achieved lower levels of fat mass (%) at both post-intervention and 3 years after intervention, in comparison with the other groups.

## 1. Introduction

Negative energy balance is required for weight loss and early maintenance phase [1]. Behavioral interventions, including reduction in energy intake (diet) and increase in energy expenditure (exercise/physical activity), result in weight loss of up to 10% of initial body weight within 6 months [2]. Results from a systematic review of 80 studies [3] showed that weight loss tends to reach a plateau, ranging between 5.0 and 8.5 kg (5–9% initial body weight) after 6 months’ treatment, gradually increasing to 3.0–5.0 kg (3–6% initial body weight) after 48 months. In agreement with these results, Curioni and Lourenco compared, after a 1-year follow-up, a weight loss intervention based on diet and exercise with that based on diet alone and observed a weight loss of 6.7 versus 4.5 kg, respectively [4]. Recently, a study showed that this represents a weight cycling with a consistent pattern in which people lose weight, reaching a plateau around 6 months, and a weight gain follows [5].

Scientific evidence suggests that a combination of dietary modification and exercise is the most effective behavioral approach for achieving weight loss. Findings from a meta-analysis showed an extra weight loss of about 1.3 kg achieved with exercise associated with a diet intervention in a 24-month follow-up [6], while in another meta-analysis, the additional effect of exercise was slightly higher, about 1.8 kg after 20 months [3]. Few studies have compared the effects of strength and endurance training or its combination on long-term weight regain [7,8], showing no differences between groups.

The long-term change in body weight has been widely studied. However, studies comparing different types of exercise-based interventions have not shown a consistent effect of training on long-term body composition maintenance. Therefore, in the present study we determined the effect on body composition (body weight, body mass index (BMI), fat mass (FM), and fat-free mass (FFM)) of different exercise-based interventions (strength, endurance, combined endurance and strength, and physical activity recommendation group (control group)) combined with diet in overweight and obese adults, both at the end of a 6-month weight loss program and 3 years after the intervention.

## 2. Materials and Methods

### 2.1. Design

The present randomized control trial (ClinicalTrials.gov ID: NCT01116856) followed the ethical guidelines of the Declaration of Helsinki. The Institutional Review Board of the La Paz University Hospital (PI-643) reviewed and approved the study design and research protocol. Details of the study’s theoretical rationale, protocol, and intervention are described elsewhere [9].

### 2.2. Patient and Public Involvement

**Figure 1:**
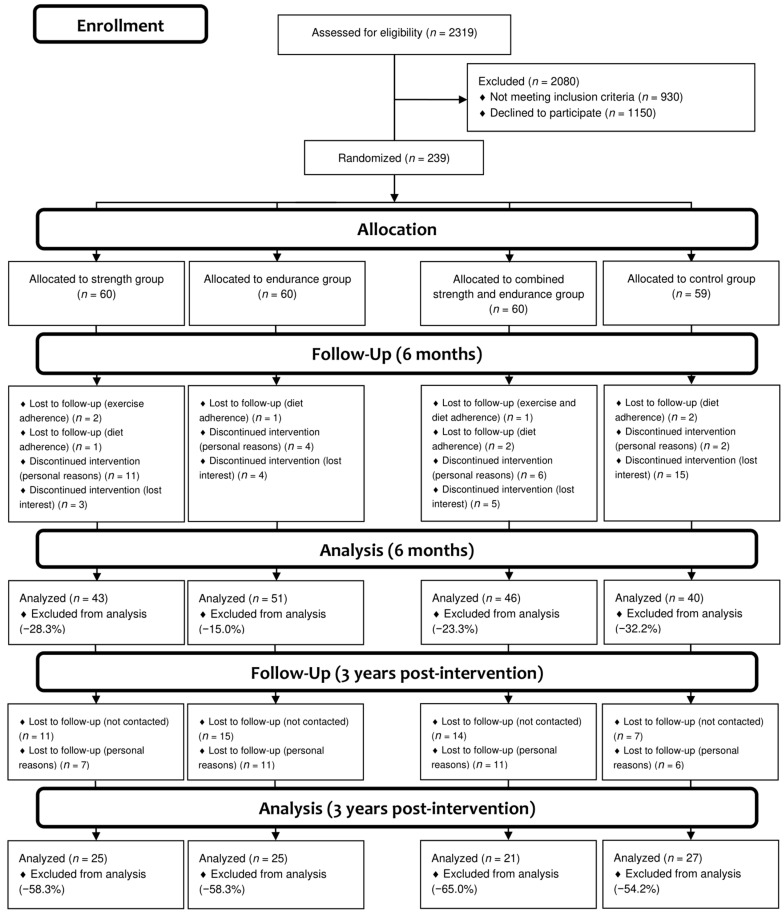
Participants’ flow diagram.

A total of 2319 potential participants (recruited through several advertisement campaigns) were informed about the nature of the study, and those who were 18 to 50 years old, had a body mass index (BMI) between 25 and 34.9 kg/m^2^, were nonsmokers, were sedentary, and had glucose values <5.6 mmol/L (<100 mg/dL) were invited to participate in this study. Women were required to have regular menstrual cycles. The 239 eligible participants who were willing to participate provided written informed consent prior to joining the study, and were randomly assigned to the groups through computer generation (Figure 1).

### 2.3. Procedures

This study consisted of a 6-month intervention based on diet and exercise. The participants entered the study in two waves, one of overweight participants and the other of obese participants. Each wave was split into four randomly assigned groups, stratified by age and sex: strength group (S), endurance group (E), combined strength and endurance group (SE), and control group (C). During the study, the participants were examined during three visits: at baseline (0 week), at the final of the intervention program (24 weeks), and after 3 years of the follow-up period of the post-intervention program. During the follow-up period, all the participants were under a free-living condition, and they were required to report about their body weight and their nutritional and physical activity behaviors every 6 months. In addition, a third face-to-face measurement took place 3 years after the intervention period.

Before the intervention started, total daily energy expenditure (TDEE) was assessed by a SenseWear Pro3 Armband™ accelerometer (BodyMedia, Pittsburgh, PA, USA), and the energy and nutritional contents of the food consumed were subsequently calculated using the DIAL software (Alce Ingeniería, 2004). Then, the negative energy balance was calculated. Moreover, the subjects of the study were required to report the type, duration, and intensity of any physical activity, as well as the amount of any possible food intake during the intervention period by recording everything in a notebook daily. Adherence to diet was calculated as the estimated kcal of the diet divided by the real kcal intake in percentage ((estimated kcal of diet/real kcal intake) × 100), 100% being the highest adherence to it, following a similar methodology used previously [10]. Furthermore, adherence to exercise was calculated by the number of sessions completed against the theoretical sessions ((sessions performed/total sessions) × 100). Adherence over 90% of the training sessions and adherence to diet over 80% were required for inclusion in the analysis.

### 2.4. Diet Intervention

Hypocaloric diets (25–30% less energy than TDEE) were prescribed individually by expert dieticians for the 22-week intervention period. Some 29–34% of energy came from fat, 50–55% from carbohydrates, and 20% from protein, according to the Spanish Society of Community Nutrition’s recommendations [11], to achieve the body composition benefits observed in different studies and examined in a meta-analysis [12].

### 2.5. Exercise Intervention

All exercise training groups (strength, endurance, and combined SE groups) followed an individualized training program, which consisted of three-times-per-week exercise sessions for 22 weeks, carefully supervised by certified personal trainers. Each training session included a 5 min aerobic warm-up, the session routine, and a 5 min cooldown and stretching exercise. Strength session routines consisted of eight exercises (i.e., shoulder press, squat, barbell row, lateral split, bench press, front split, biceps curl, and French press for triceps). Endurance session routines consisted of self-selected running, cycling, or elliptical. For the combined strength and endurance group, a combination of cycle ergometry, treadmill, or elliptical intercalated with squat, row machine, bench press, and front split was carried out. The volume and intensity of the three training groups were increased progressively. During the adaptation period (i.e., weeks 1–4), the subjects were taught the different exercise routines. During weeks 5 to 8, the exercises were carried out at an intensity of 50% of 15 repetition maximums (RM) and heart rate reserve (HRR), and the subjects performed 2 laps of the circuit (51 min and 15 s in total). During weeks 9 to 14, the intensity was increased to 60% of 15RM and HRR. Finally, during weeks 15 to 24, the volume was increased to 3 circuit laps instead of 2 (64 min in total). In addition, 5 min recovery periods were established between the circuit laps. The S and SE participants performed 15 repetitions (45 s) for each exercise, including a rest period of 15 s between repetitions. Full details of the different protocols developed by the groups are described elsewhere [9].

### 2.6. Control Group

The participants from the control group followed the dietary intervention and respected the recommendations about physical activity from the American College of Sports Medicine (ACSM) [13]. However, this activity was not supervised, and they were free to do it daily.

### 2.7. Outcome Measures

Overweight and obesity were defined by a BMI of 25 to 29.9 kg/m^2^ and 30 kg/m^2^ or greater, respectively. Body fat percentage was further dichotomized based on standard clinical definitions for men (normal fat < 25%, overfat ≥ 25%) and women (normal fat < 30%, overfat ≥ 30%) [14].

### 2.8. Primary Outcomes

Body composition (fat mass and fat-free mass) was assessed by dual-energy X-ray absorptiometry (DXA, GE Encore 2002, version 6.10.029) (GE Lunar Prodigy; GE Healthcare, Madison, WI, USA). Body weight was measured in kilograms with a Tanita scale (TANITA BC-420MA, Biológica Tecnología Médica SL, Barcelona, Spain). Height was measured using a SECA stadiometer (80–200 cm range). The BMI was calculated as (body weight (kg)/(height (m))^2^). For the evaluation of the primary outcomes, the participants were cited, in each measurement moment, between 7 and 10 a.m., after a fasting period of 9 h, to ensure the standardization and accuracy of the evaluations. In addition, the DXA was calibrated every day prior to the measurement of the participants.

### 2.9. Secondary Outcomes

Short-term success was defined as a body weight loss equal to or greater than 10% of the initial body weight [15], and long-term maintenance success was determined as a maintenance equal to or greater than 5% [16].

### 2.10. Statistical Analyses

Data were analyzed using PASW Statistics version 18.0 for Windows (SPSS Inc., Chicago, IL, USA). Values of *p* below 0.05 were considered statistically significant, and where possible, partial eta squared (η^2^) was reported for the effect size of the comparisons. Primary analysis (per-protocol analysis) was performed in participants with complete data on the study outcomes at the three measurement points (pre-intervention, post-intervention, and 3 years after intervention). To make the most robust analysis, we also conducted intention-to-treat analysis; if the outcome value was missing for the participant, the last valid observation was carried forward assuming no change in that outcome variable. We conducted a chi-square test to compare the prevalence of dropouts at post-intervention and 3 years after the intervention across groups. Differences in the outcome variables (body weight, BMI, FM, and FFM) between dropouts and participants with available data on the study outcome 3 years after intervention were determined with an analysis of covariance (ANCOVA), after adjusting for sex, age, and the corresponding value of the study outcome at pre-intervention. Moreover, we conducted an ANCOVA to compare the outcome variables (body weight, BMI, FM, and FFM) between overweight and obese participants at post-intervention, after adjusting for sex, age, and the corresponding value of the study outcome at pre-intervention.

We used a two-factor (group and time) ANCOVA with repeated measures to assess the intervention effects on the outcome variables (body weight, BMI, FM, and FFM) after adjusting for sex and age. For each variable, we reported the *p*-value corresponding to the group (between subjects), time (within subjects), and interaction (group × time) effects. We calculated the *p*-value for within-group differences separately by group when a significant interaction effect was present. Moreover, we conducted an ANCOVA to compare mean differences in the study outcome (post-intervention minus pre-intervention and 3 years minus pre-intervention), where the variable group was inserted as a fixed factor; sex, age, and the corresponding baseline value of the study outcome were entered as covariates, and the outcome variables were entered as dependent variables. Multiple comparisons were made with a Bonferroni post hoc test.

We conducted a chi-square test to compare the prevalences of normal weight, overweight, and obese participants, as well as the prevalence of normal fat or overfat participants at pre-intervention, post-intervention, and 3 years after intervention among the groups. Moreover, a chi-square test was conducted to compare the frequency of participants who maintained, decreased, or increased their weight status (normal weight, overweight, and obese according to BMI, and normal fat or overfat according to fat mass) after the exercise intervention (post-intervention minus pre-intervention) and 3 years later (3 years minus post-intervention, and 3 years minus pre-intervention) among the groups. Finally, we determined the percentage of “successful weight maintainers.”

## 3. Results

A total of 239 participants initially participated in the study, and 180 completed it (75.3%), with 59 dropouts for different reasons: low exercise adherence, 3 (5.0%); low diet adherence, 6 (10.2%); personal reasons, 23 (39.0%); and lost interest, 27 (45.8%). After a 3-year follow-up period, 98 (41%) participants attended the last visit programed in the study (Figure 1). There was no association in the prevalence of dropouts among the groups at post-intervention (χ^2^ = 5.309, *p* = 0.150) and 3 years after intervention (χ^2^ = 1.468, *p* = 0.690). Moreover, there were no significant associations in the study outcomes at post-intervention between dropouts and participants who remained in the study (all *p* > 0.1, data not shown).

No significant interactions between groups and BMI status were observed (all *p* > 0.05) for the study outcomes, showing a tendency toward significance for the variable FM (%), the loss being greater for the combined strength and endurance group than the others for the participants who were overweight at the beginning and not for the obese participants (data not shown). Table 1 shows the values of the primary outcomes at pre-assessment, post-assessment, and 3 years’ assessment by group. A significant interaction effect (group × time) was observed for the fat mass percentage (*p* = 0.048). Fat mass percentage decreased in all groups after the 6-month intervention period (all *p* < 0.001). However, it was regained 3 years after the intervention period to similar values as during pre-intervention, except for the combined strength and endurance group, who kept it reduced (*p* < 0.001). No significant interaction effect (group × time) was observed in body weight, body mass index, fat mass (kg), and fat-free mass after adjusting for multiple comparisons.

**Figure 2:**
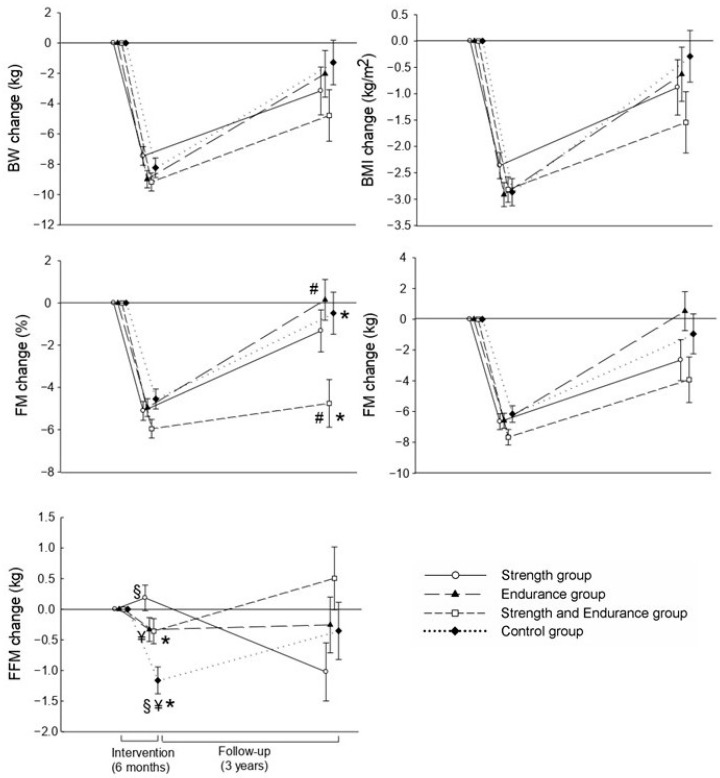
Body composition changes between post-intervention and pre-intervention (6 months’ intervention) and 3 years after intervention and post-intervention (3 years’ follow-up), adjusted by sex, age, and the corresponding baseline value of the study outcome. Common superscripts indicate a significant difference between groups (all *p* ≤ 0.05). BW: body weight; BMI: body mass index; FM: fat mass; FFM: fat-free mass.

Figure 2 shows the body composition changes between post-intervention minus pre-intervention, 3 years minus post-intervention, and 3 years minus pre-intervention. The combined strength and endurance group achieved greater reductions in fat mass percentage compared with the endurance and control groups (*p* = 0.008 and *p* = 0.003, respectively) after 3 years. This tendency was observed between the SE and S groups; however, the statistical difference was borderline (*p* = 0.05). The control group had a higher reduction in FFM compared with the training groups (*p* < 0.05) after the intervention period.

Table 2 shows the prevalences of normal weight, overweight, and obese participants, as well as the prevalence of normal fat or overfat participants, at pre-intervention, post-intervention, and 3 years after intervention by group. There was an association in BMI categories among the groups (χ^2^ = 12.925, *p* = 0.044) at post-intervention; the combined strength and endurance group had significantly higher percentage of normal weight participants than expected (standardized residual: 2.4). There was an association in BMI categories among the groups 3 years after intervention (χ^2^ = 16.778, *p* = 0.010); the combined strength and endurance group had significantly higher percentage of normal weight participants than expected (standardized residual: 3.0). In the fat mass categories, there was an association 3 years after intervention among the groups (χ^2^ = 13.224, *p* = 0.004); the combined strength and endurance group had significantly higher normal fat (%) participants than expected (standardized residual: 2.9).

**Figure 3:**
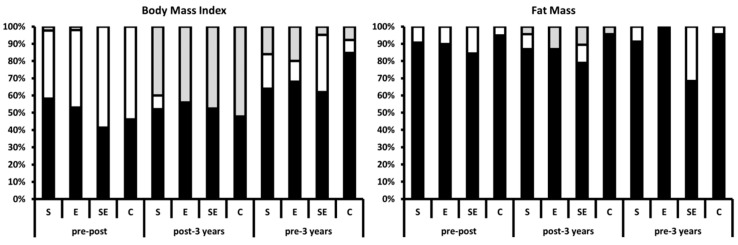
Frequency of participants who maintained (black column), decreased (white column), or increased (gray column) their body weight status according to their body mass index (left panel) and fat mass (right panel) by group. Pre–post: changes between pre-intervention and post-intervention (6 months); post–3 years: changes between post-intervention and 3 years (3 years’ follow-up); pre–3 years: changes between pre-intervention and 3 years after intervention (3 years and 6 months).

Figure 3 shows the frequency of participants who maintained, decreased, or increased their body weight status according to their BMI and fat mass by group. There were no differences in BMI categories among the groups at pre-intervention, post-intervention, and 3 years after intervention (all *p* > 0.05). However, there was a significant association among the groups in the fat mass categories 3 years after intervention (χ^2^ = 13.224, *p* = 0.004); the SE group had significantly higher percentage of normal fat participants than expected (standardized residual: 2.9).

Eighty-five out of 180 participants (47.2%) reduced their body weight after intervention by higher than 10%. Considering the strength, endurance, combined strength and endurance groups and the control, the distribution of these participants was 16 (18.8%), 28 (32.9%), 25 (29.5%), and 16 (18.8%), respectively (χ^2^ = 4.766, *p* = 0.190). After 3 years, 32 out of 98 participants (32.7%) were able to maintain their body weight at values lower than 5%. Considering the strength, endurance, combined strength and endurance groups and the control, the distribution of these participants was 9 (28.1%), 7 (21.9%), 10 (31.3%), and 6 (18.7%), respectively (χ^2^ = 3.848, *p* = 0.278).

Intention-to-treat analysis did not show any significant changes from the results obtained with per-protocol analysis (data not shown).

## 4. Discussion

The main finding of this study was that the different exercise treatments developed during the weight loss program, in combination with caloric restriction, obtained similar results for body weight at post-intervention and 3 years after intervention. However, differences were observed between groups regarding fat mass percentage. It is important to highlight, as shown in Figure 3, that the combined strength and endurance group was the one that showed the highest number of participants who reduced their BMI or the percentage of fat mass 3 years after intervention.

In weight loss programs that exceed 16 weeks of intervention, a flattening occurs in the weight loss curves [17], and if the maintenance period is not continued by a hypocaloric diet, then body weight regain occurs. Moreover, large and persistent increases in physical activity may be required for long-term maintenance of lost weight [1,18]. A review concluded that weight loss tends to reach a plateau, ranging between 5.0 and 8.5 kg (5–9% initial body weight) after 6 months’ treatment, gradually increasing to 3.0–5.0 kg (3–6% initial body weight) after 48 months [19]. The results obtained from our study are in accordance with previous literature, reaching losses of about 10% from the initial body weight at the end of the intervention [19]. We observed a weight loss of about 8.6 kg (9.8% of the initial body weight) at 6 months. Three years after intervention, we observed a weight loss of about 2.8 kg (3.2% of the initial body weight). It is important to highlight that no differences were discovered among the groups at the two-time points. These results are in contrast with those of a recent study that discovered body weight regains of 52% ± 38% and 89% ± 54% of initial body weight lost at 1 and 2 years’ follow-up, respectively [20]. Probably, the behavioral character of our intervention may have facilitated the maintenance of body weight after completion, compared with the previous study, which included a very low-calorie diet.

In this study, overall weight loss was similar, but body composition changes were much better after 22 weeks of diet plus physical exercise (considering the strength, endurance, and combined strength and endurance groups), compared with the control group. While the fat-free mass decreased in the control group, the rest of the groups maintained it. Evidence of the beneficial effects of physical exercise on FFM has also been described in another study [21], especially the combination of endurance and resistance exercises [22]. Fat-free mass (FFM) is the main factor that accounts for the magnitude of resting metabolism [23]. Therefore, any diet or exercise interventions that can maintain FFM or at least attenuate its decline following weight loss could have significant effects on the total energy balance [24].

In the present study, we compared three types of exercises, obtaining similar results for body weight during intervention and at 3 years’ follow-up. However, similar to the findings of a study previously published by Sillanpaa et al. [25], the combined strength and endurance group was the one that reported better results for FM change (%) at post-intervention and even 3 years after. Sillanpaa et al. [25] affirmed that these changes might be due to differences in the amount and intensity of training and the specific exercises performed. Moreover, current scientific evidence supports that the endurance and strength exercise combination within the same training session might be assumed by the participants to be more motivating, decreasing even the level of exertion perceived [26]. This agrees with the results of previous studies in which exercise self-efficacy was presented as a mediator for weight loss [27,28]. Therefore, the combination of strength and endurance exercises within the same training session could help participants initiate a healthier lifestyle.

Wing [29] concluded that weight regain was approximately 43% across a 40-month period following initial weight loss, and similar results were reported by Perri and Corsica [30]. In this study, similar results were obtained 3 years after intervention. However, analysis based on randomized group assignment did not indicate a favorable contribution of exercise to weight loss maintenance as previously reported by Jakicic et al. [31]. Studies with similar duration have shown comparable results, with physical activity initially increasing before gradually decreasing over time [32]. Thus, the inability to sustain weight loss appears to mirror the inability to sustain physical activity [31].

However, the present study has some limitations. First, the percentage of participants who completed the follow-up study was relatively low. It is worth noting that this rate of participation is comparable to that of other long-term studies with 3–4 years’ follow-up. The attrition rate was 46.2%, in accordance with two previous studies with a similar follow-up time frame, and the average attrition rates were 51.8% and 53.0%, respectively [33]. Therefore, our understanding of weight loss and regain is complicated by high attrition rates of up to 90% in obesity treatment trials.

## 5. Conclusions

In conclusion, our findings indicate that the four types of treatments had similar results in body composition changes at the end of the intervention period, with the exception of FFM. Only the exercise groups (strength, endurance, and combined strength and endurance groups) maintained FFM during the caloric restriction. Therefore, it is suggested that physical exercise be included in weight loss programs based on caloric restriction, with the objective of maintaining fat-free mass, beyond the proposed physical activity recommendations. In addition, it is suggested that this exercise be combined (strength and endurance) to maintain FM (%) and reduced BMI 3 years after the end of the intervention, since only the combined strength and endurance group maintained the FM (%) reduced 3 years after the end of the intervention.

## Figures and Tables

**Table 1:** Values of body composition variables at pre-intervention, post-intervention, and 3 years after. Presented as mean (standard error of mean) at 95% CI.

	Group	Pre (95% CI)	Post (95% CI)	3 Years (95% CI)	*p*-Group	η^2^	*p*-Time	η^2^	*p*-Interaction	η^2^
Body Weight (kg)	S	92.3 (2.0) (88.3–96.3)	83.7 (2.0) (79.8–87.65)	89.1 (2.5) (84.1–94.1)	0.019	0.102	0.139	0.024	0.435	0.029
	E	85.9 (2.0) (81.8–89.9)	77.0 (2.0) (73.0–80.9)	83.8 (2.5) (78.8–88.9)						
	SE	83.3 (2.2) (79.0–87.7)	74.4 (2.2) (70.1–78.7)	78.6 (2.7) (73.1–84.1)						
	C	86.8 (1.9) (82.9–90.6)	79.0 (1.9) (75.2–82.7)	85.5 (2.4) (80.7–90.3)						
Body Mass Index (kg/m^2^)	S	31.6 (0.5) (30.6–32.6)	29.0 (0.5) (27.9–30.0)	30.7 (0.7) (29.3–32.1)	0.002	0.150	0.102	0.029	0.382	0.033
	E	30.4 (0.5) (29.5–31.4)	27.3 (0.5) (26.3–28.4)	29.8 (0.7) (28.4–31.2)						
	SE	29.0 (0.5) (27.9–30.0)	25.9 (0.6) (24.7–27.1)	27.4 (0.8) (25.9–28.9)						
	C	30.7 (0.5) (29.8–31.6)	28.1 (0.5) (27.1–29.1)	30.4 (0.7) (29.1–31.8)						
Fat Mass (%)	S	41.5 (0.9) (39.8–43.3)	36.1 ^†^ (1.0) (34.0–38.1)	40.1 ^§^ (1.3) (37.6–42.7)	0.001	0.187	0.586	0.004	0.017	0.116
	E	40.5 (0.9) (38.7–42.3)	35.2 ^†^ (1.0) (33.1–37.2)	40.6 ^§^ (1.3) (38.1–43.1)						
	SE	38.1 (1.0) (36.1–40.0)	31.0 ^¶,◊,#,†^ (1.1) (28.8–33.3)	33.6 ^¶,◊,#,¥^ (1.4) (30.8–36.3)						
	C	41.5 (0.9) (39.7–43.2)	36.6 ^†^ (1.0) (34.5–38.6)	40.9 ^§^ (1.3) (38.4–43.4)						
Fat Mass (kg)	S	36.3 (1.3) (33.8–38.9)	28.7 (1.3) (26.1–31.2)	33.9 (1.9) (30.1–37.6)	0.005	0.144	0.282	0.015	0.238	0.047
	E	33.0 (1.3) (30.4–35.5)	26.2 (1.3) (23.7–28.8)	33.4 (1.9) (29.6–37.1)						
	SE	29.9 (1.4) (27.1–32.7)	22.0 (1.4) (19.2–24.9)	25.8 (2.1) (21.7–29.9)						
	C	34.0 (1.3) (31.5–36.6)	27.8 (1.3) (25.2–30.3)	33.1 (1.9) (29.4–36.8)						
Fat-Free Mass (kg)	S	51.7 (1.1) (49.5–53.8)	51.5 (1.1) (49.3–53.7)	50.7 (1.2) (48.3–53.1)	0.198	0.055	<0.001	0.144	0.205	0.054
	E	48.7 (1.1) (46.6–50.9)	48.5 (1.1) (46.3–50.7)	48.3 (1.2) (45.9–50.7)						
	SE	49.1 (1.2) (46.7–51.5)	49.1 (1.2) (46.6–51.5)	49.6 (1.3) (47.0–52.3)						
	C	48.5 (1.1) (46.4–50.7)	47.7 (1.1) (45.4–49.9)	48.1 (1.2) (45.7–50.5)						

Note: S: strength training group; E: endurance training group; SE: combined strength and endurance training group; C: control group. ^¶^: SE–S differences; ^#^: SE–C differences; ^◊^: SE–E differences; ^†^: post–pre differences; ^§^: 3 years–post differences; ^¥^: 3 years–pre differences. Significant statistical level set at α = 0.05.

**Table 2:** Prevalences of normal weight, overweight, and obese participants, as well as prevalence of normal fat or overfat participants at pre-intervention, post-intervention, and 3 years after intervention by group.

			Pre				Post				3 Years			
		Group	Freq. (%)		Mean (SD)		Freq. (%)		Mean (SD)		Freq. (%)		Mean (SD)	
Body Mass Index (kg/m^2^)	Normal Weight (20–24.9)	S	-		-		2	(7.4)	23.7	(0.3)	2	(18.2)	24.2	(0.5)
		E	-		-		6	(22.2)	23.5	(0.8)	-		-	
		SE	-		-		13	(48.2)	23.9	(0.7)	7	(63.6)	23.6	(1.2)
		C	-		-		6	(22.2)	23.6	(1.2)	2	(18.2)	24.3	(0.6)
	Overweight (25–29.9)	S	21	(23.9)	28.8	(1.1)	30	(27.3)	27.7	(1.2)	9	(23.6)	28.2	(1.1)
		E	26	(29.5)	28.4	(1.3)	36	(32.7)	27.3	(1.4)	13	(34.2)	27.5	(1.1)
		SE	23	(26.1)	27.8	(1.2)	20	(18.2)	27.3	(1.4)	8	(21.1)	27.3	(1.2)
		C	18	(20.5)	28.3	(1.3)	24	(21.8)	27.4	(1.1)	8	(21.1)	27.9	(1.5)
	Obese (30–34.9)	S	21	(23.1)	32.5	(1.6)	9	(24.3)	31.9	(1.9)	14	(29.2)	33.2	(2.1)
		E	25	(27.5)	32.4	(1.8)	9	(24.3)	31.2	(0.8)	12	(25.0)	32.5	(3.1)
		SE	23	(25.3)	32.9	(2.0)	11	(29.7)	32.5	(1.9)	6	(12.5)	32.0	(1.7)
		C	22	(24.1)	32.5	(1.7)	8	(21.7)	32.2	(2.3)	16	(33.3)	32.8	(2.3)
Fat Mass (%)	Normal Fat (♂ < 25%; ♀ < 30%)	S	-		-		4	(22.2)	22.0	(1.7)	2	(22.2)	22.9	(0.9)
		E	-		-		5	(27.8)	24.4	(0.3)	-		-	
		SE	-		-		7	(38.9)	21.7	(3.6)	6	(66.7)	19.2	(5.8)
		C	-		-		2	(11.1)	22.5	(2.1)	1	(11.1)	23.9	
	Overfat (♂ < 25%; ♀ < 30%)	S	43	(23.9)	41.2	(6.2)	39	(24.7)	37.5	(5.7)	21	(26.3)	42.1	(5.9)
		E	51	(28.3)	41.3	(6.0)	44	(27.8)	37.8	(5.6)	24	(30.0)	40.0	(7.4)
		SE	46	(25.6)	40.5	(7.3)	38	(24.1)	36.7	(7.2)	13	(16.3)	38.0	(7.9)
		C	40	(22.2)	41.2	(5.4)	37	(23.4)	37.2	(5.8)	22	(27.4)	43.0	(6.0)

Note: S: strength training group; E: endurance training group; SE: combined strength and endurance training group; C: control group.

## Data Availability

The data presented in this study are available on request from the corresponding author.
